# Ischemic heart disease mortality trends and disparities in US adults from 1999 to 2023: a CDC WONDER analysis

**DOI:** 10.3389/fcvm.2026.1883973

**Published:** 2026-09-18

**Authors:** Jia Li, Bolin Wang, Xuezhen Xuan, Fan Zhang, Sichong Qian, Haiyang Li

**Affiliations:** Department of Cardiac Surgery, Beijing Anzhen Hospital, Capital Medical University, Beijing, China

**Keywords:** AAMR, CDC WONDER, COVID-19 pandemic, IHD, the United States

## Abstract

**Background:**

Ischemic heart disease (IHD) remains a leading cause of mortality in the United States, yet it remains unclear whether postpandemic recovery patterns differ across population subgroups.

**Methods:**

Using CDC WONDER data (1999–2023), we analyzed IHD deaths among US adults aged ≥25 years. Age-adjusted mortality rates (AAMRs) were stratified by sex, race/ethnicity, urbanization, and region; crude mortality rates (CMRs) by age. Urban–rural analyses were restricted to 1999–2020. Joinpoint regression estimated temporal trends, and tests of parallelism assessed between-group differences.

**Results:**

Among 10,200,362 IHD deaths, overall AAMRs declined from 300.8 to 127.07 per 100,000 (average annual percent change, AAPC = −3.51; *p* < 0.001), with the fastest decline during 2021–2023 (APC = −5.59). Males had higher AAMRs than females (245.58 vs. 138.69; *p* < 0.001). Non-Hispanic (NH) Black individuals had the highest AAMR (217.58). The steepest age-specific CMR decline occurred at 75–84 years (AAPC = −4.04). Metropolitan areas had lower AAMRs than non-metropolitan areas (189.07 vs. 212.60; *p* < 0.001). Pairwise tests confirmed significant trend differences across most subgroups (all *p* < 0.05), except NH Other vs. NH White (*p* = 0.396).

**Conclusions:**

Despite sustained IHD mortality declines, significant disparities persist across demographic and geographic subgroups, supporting targeted prevention for high-risk populations.

## 1 Background

Ischemic heart disease (IHD), also termed coronary artery disease (CAD) and atherosclerotic cardiovascular disease (ASCVD), is clinically characterized by myocardial infarction and ischemic cardiomyopathy. Non-fatal IHD increasingly leads to chronic disability and impaired quality of life among patients (1). Its primary pathological mechanism is atherosclerosis—an arterial inflammatory disorder linked to lipid deposition and metabolic dysregulation driven by multiple risk factors (2).

Cardiovascular disease (CVD) accounts for approximately one-third of global deaths (3), with IHD being the most prevalent subtype (4). As a leading cause of mortality and morbidity worldwide, IHD imposes a high disease burden and long-term impacts on quality of life, highlighting its status as a critical public health challenge (5). Globally, IHD was associated with 182 million disability-adjusted life years (DALYs) in 2019 (6), ranking second among all age groups (7.2% of the total DALYs) and first in adults aged 50–74 years (11.8%) and ≥75 years (16.2%) (7). In the United States, IHD deaths have risen substantially, contributing to soaring healthcare expenditures and threatening 21st-century sustainable development. With the United Nations 2030 Agenda for Sustainable Development approaching, achieving the Sustainable Development Goal (SDG)—including a one-third reduction in premature non-communicable disease (NCD) mortality, partially attainable by mitigating IHD burden (8)—is imperative. Thus, monitoring and predicting IHD burden remains crucial.

IHD is a major driver of CVD-related morbidity and mortality, and its prevention and treatment remain global challenges.

Previous studies have examined IHD mortality trends in the United States through 2020; however, the period since 2020 represents a critical inflection point. The COVID-19 pandemic disrupted healthcare delivery, altered mortality patterns, and disproportionately affected individuals with pre-existing cardiovascular conditions. Whether IHD mortality trends have returned to their prepandemic trajectories and whether all population subgroups have experienced similar recoveries remain unknown. Using the most recent data of the Centers for Disease Control and Prevention Wide-Ranging Online Data for Epidemiologic Research (CDC WONDER) through 2023, this study aimed to (1) characterize overall IHD mortality trends over the full 25-year period; (2) identify whether post-2020 recovery patterns differed by sex, race/ethnicity, and geographic region; and (3) determine which subgroups remain at highest risk in the postpandemic era.

## 2 Materials and methods

### 2.1 Data sources

Data for this study were obtained from CDC WONDER. CDC WONDER is a comprehensive death certificate database covering all 50 US states and the District of Columbia. Mortality data extracted from death certificates and coded using the International Classification of Diseases, Tenth Revision (ICD-10), align with current literature on national mortality trend analyses (9). IHD was defined by ICD-10 codes I20–I25. As CDC WONDER provides anonymized, publicly available data, this study was exempt from institutional review board (IRB) approval.

### 2.2 Data extraction

Included cases were US adults aged ≥25 years with IHD recorded as the underlying cause of death between 1999 and 2023. Although rare IHD-related deaths can occur in individuals younger than 25 years, we adopted this age cutoff in accordance with well-established cardiovascular mortality surveillance frameworks, given that population-level IHD burden and demographic disparities predominantly manifest among adults aged 25 years and older (10). IHD subtypes corresponded to the following ICD-10 codes: I20 (angina pectoris), I21 (acute myocardial infarction), I22 (subsequent myocardial infarction), I24 (other acute IHD), and I25 (chronic IHD). Data were stratified by sex (male, female), 10-year age groups (25–34, 35–44, 45–54, 55–64, 65–74, 75–84, 85+ years), and race/ethnicity [non-Hispanic (NH) White, NH Black, Hispanic, NH Other], and urban–rural status was categorized according to the National Center for Health Statistics Urban-Rural Classification Scheme. Urban–rural analyses were limited to the period 1999–2020, given that CDC WONDER data are available only for this time frame. Geographic regions were defined using US Census Bureau census classifications (Northeast, Midwest, South, West) (11).

### 2.3 Data analysis

A total of 10,200,362 cases were included. Crude mortality rate (CMR) and age-adjusted mortality rate (AAMR) per 100,000 population were calculated. CMR was computed as the number of IHD-related deaths per year divided by the corresponding US population. AAMR was standardized to the 2000 US standard population to account for age-related differences in population composition (12).

Temporal trends in AAMR and CMR were assessed using the Joinpoint Regression Program (Version 5.3.1, National Cancer Institute). This tool fits log-linear regression models (ln[rate] = x*β*) with time-varying joinpoints to identify time points at which mortality trends changed significantly. The maximum number of joinpoints was set to 4, with a minimum of 2 observations from a joinpoint to either end of the data, to avoid overfitting. The optimal model for each subgroup was determined using the data-driven Weighted Bayesian Information Criterion (WBIC) method. The statistical significance of identified trends was assessed using Monte Carlo permutation tests with 4,499 permutations, with an overall significance level of *α* = 0.05. Annual percent change (APC) and average annual percent change (AAPC) with their corresponding 95% confidence intervals (CIs) were reported for each identified trend segment and for the full study period, respectively. Confidence intervals for the final selected model were calculated using the empirical quantile method with 5,001 resamples. Within-group trends were considered statistically significant if the APC or AAPC regression slope differed significantly from zero (two-tailed t-test, *p* < 0.05).

To evaluate whether IHD mortality time trends differed significantly between population subgroups, pairwise comparisons were performed using the Joinpoint Regression Program's built-in pairwise comparison function. Specifically, tests of parallelism were conducted to assess whether the slopes (APC/AAPC) of IHD mortality trends differed significantly between any two subgroups defined by sex, race/ethnicity, census region, and urbanization level. For pairwise parallelism tests between two distinct subgroups, the *K*_max_ adopted for each comparison was inherited from the WBIC-selected optimal joinpoint quantity of each corresponding individual subgroup. This universal rule applies to all stratified analyses (sex, race/ethnicity, urban–rural status, and census region). Consequently, pairwise comparisons involving the same single group may adopt different *K*_max_ values. For illustration, the comparison between Hispanic and NH Other subgroups used *K*_max_ = 2, whereas comparisons of Hispanic vs. NH Black and Hispanic vs. NH White both adopted *K*_max_ = 4. The analysis was based on a Monte Carlo permutation procedure with 4,500 permutations, and a two-sided *p*-value <0.05 was considered statistically significant. Rejection of the null hypothesis of parallelism indicates that the temporal trends differed significantly between the compared groups. Of note, this software-driven approach may yield different maximum joinpoint specifications across pairwise contrasts, which introduces a potential risk for spurious rejection of the null hypothesis of parallelism; this caveat should be considered when interpreting pairwise testing outputs.

## 3 Results

### 3.1 Overall

A total of 10,200,362 IHD deaths were recorded among US adults aged ≥25 years between 1999 and 2023. The AAMR declined from 300.8 per 100,000 in 1999 to 127.07 per 100,000 in 2023, with an overall AAMR of 185.22 (95% CI: 184.65–185.79) and an AAPC of −3.51 (95% CI: −3.99 to −3.03; *p* < 0.001). A comprehensive summary of this study is illustrated in Figure 1 and Table 1. Joinpoint regression identified distinct temporal phases: 1999–2002: APC = −3.81 (95% CI: −5.13 to −2.48; *p* < 0.001); 2002–2010: APC = −5.29 (95% CI: −5.67 to −4.91; *p* < 0.001); 2010–2018: APC slowed to −2.77 (95% CI: −3.20 to −2.35; *p* < 0.001); 2018–2021: APC shifted to a non-significant slight increase (1.12; 95% CI: −2.14 to 4.49); 2021–2023: APC = −5.59 (95% CI: −8.68 to −2.39; *p <* 0.01), representing the fastest decline phase across the study period (Figure 2 and Supplementary Tables S1, S2).

**Figure 1:**
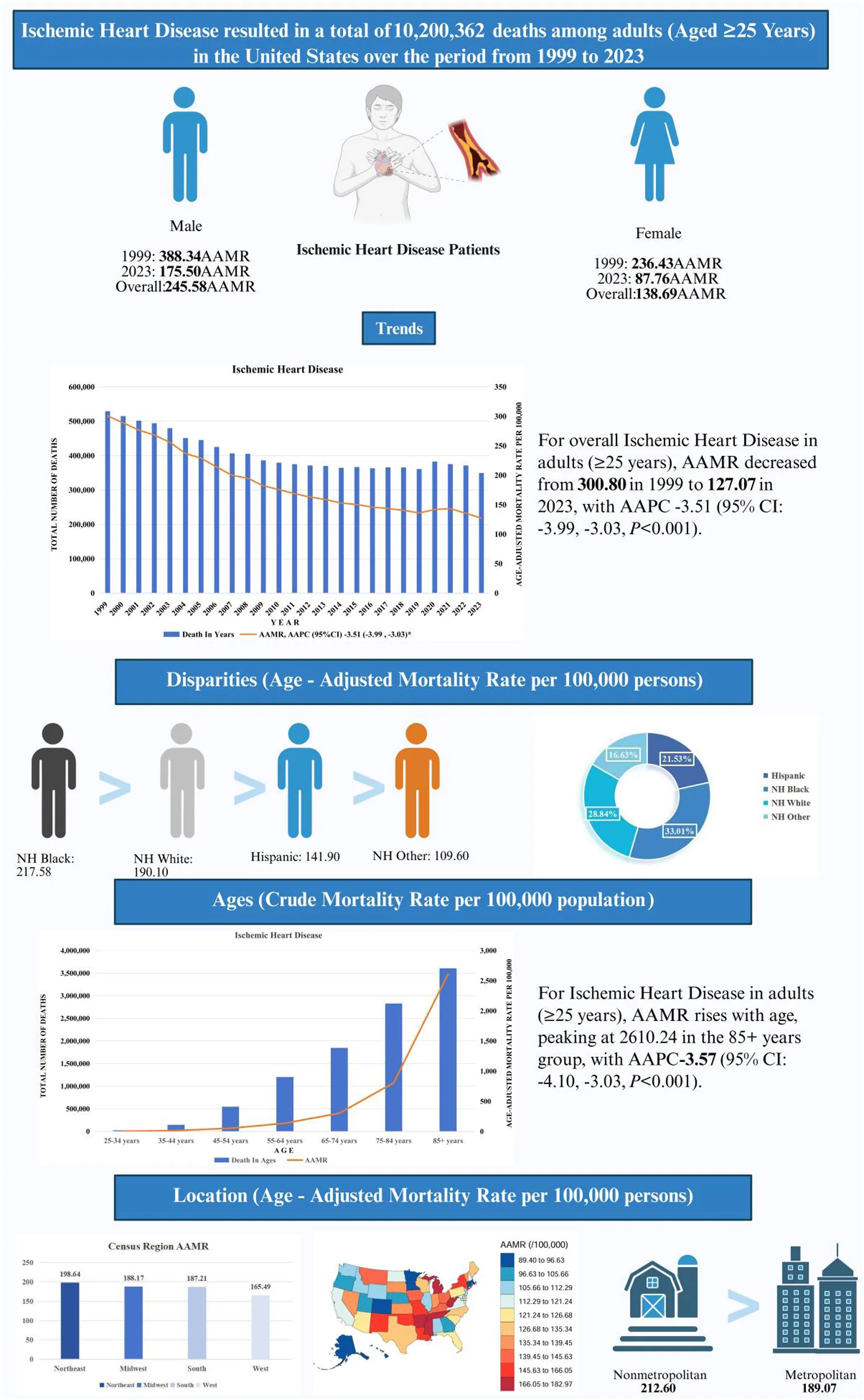
Geographic heterogeneity in ischemic heart disease mortality within the overall US population during the period 1999–2023.

**Table 1:** IHD-related mortality among US adults (aged ≥25 years) (1999–2023): deaths, AAMR, and APC.

Variable	Deaths (*n*)	Overall AAMR (95% CI)	Trend segment	Year interval	APC (95% CI)	AAPC (95% CI)
Overall	10,200,362	185.22 (184.65–185.79)	1	1999–2002	‒3.81 (−5.13 to −2.48)***	‒3.51 (−3.99 to −3.03)***
			2	2002–2010	‒5.29 (−5.67 to −4.91)***	
			3	2010–2018	‒2.77 (−3.20 to −2.35)***	
			4	2018–2021	1.12 (−2.14 to 4.49)	
			5	2021–2023	‒5.59 (−8.68 to −2.39)**	
Sex						
Female	4,601,456	138.69 (137.82–139.56)	1	1999–2002	‒3.75 (−5.19 to −2.30)***	‒4.04 (−4.61 to −3.47)***
			2	2002–2010	‒5.86 (−6.29 to −5.44)***	
			3	2010–2018	‒3.52 (−4.01 to −3.02)***	
			4	2018–2021	0.67 (−3.22 to 4.71)	
			5	2021–2023	‒6.05 (−9.77 to −2.17)**	
Male	5,598,906	245.58 (244.55–246.62)	1	1999–2010	‒4.74 (−4.97 to −4.52)***	‒3.29 (−3.80 to −2.77)***
			2	2010–2018	‒2.46 (−2.94 to −1.97)***	
			3	2018–2021	1.37 (−2.25 to 5.12)	
			4	2021–2023	‒5.32 (−8.73 to −1.78)**	
Race						
Hispanic	563,283	141.90 (139.97–143.83)	1	1999–2014	‒5.31 (−5.73 to −4.89)***	‒4.13 (−4.97 to −3.29)***
			2	2014–2021	‒0.64 (−2.21 to 0.95)	
			3	2021–2023	‒7.21 (−15.38 to 1.76)	
NH Black	1,078,856	217.58 (215.49–219.68)	1	1999–2002	‒2.53 (−3.71 to −1.32)***	‒3.51 (−3.91 to −3.10)***
			2	2002–2011	‒5.49 (−5.77 to −5.21)***	
			3	2011–2018	‒2.71 (−3.18 to −2.25)***	
			4	2018–2021	2.66 (−0.07 to 5.45)	
			5	2021–2023	‒7.59 (−10.09 to −5.01)***	
NH White	8,260,439	190.10 (189.45–190.75)	1	1999–2010	‒4.87 (−5.11 to −4.62)***	‒3.40 (−4.02 to −2.77)***
			2	2010–2018	‒2.69 (−3.25 to −2.12)***	
			3	2018–2021	1.18 (−3.20 to 5.76)	
			4	2021–2023	‒4.75 (−8.96 to −0.36)*	
NH Other	263,867	109.60 (107.43–111.78)	1	1999–2015	‒4.47 (−4.74 to −4.20)***	‒3.74 (−4.30 to −3.19)***
			2	2015–2021	‒0.63 (−2.00 to 0.77)	
			3	2021–2023	‒7.05 (−12.21 to −1.58)*	
Urban/rural						
Metropolitan	7,342,045	189.07 (188.43–189.71)	1	1999–2002	‒3.82 (−5.11 to −2.52)***	‒3.69 (−4.06 to −3.33)***
			2	2002–2010	‒5.51 (−5.89 to −5.14)***	
			3	2010–2018	‒2.93 (−3.34 to −2.51)***	
			4	2018–2020	0.90 (−2.29 to 4.20)	
Non-metropolitan	1,762,306	212.60 (211.12–214.08)	1	1999–2010	‒4.26 (−4.55 to −3.98)***	‒3.06 (−3.28 to −2.84)***
			2	2010–2020	‒1.72 (−2.10 to −1.34)***	
Region						
Northeast	2,177,581	198.64 (197.32–199.96)	1	1999–2002	‒3.56 (−5.06 to −2.03)***	‒3.86 (−4.42 to −3.29)***
			2	2002–2011	‒5.19 (−5.56 to −4.83)***	
			3	2011–2017	‒2.98 (−3.83 to −2.11)***	
			4	2017–2020	‒0.10 (−4.06 to 4.03)	
			5	2020–2023	‒5.53 (−7.50 to −3.51)***	
Midwest	2,339,054	188.17 (186.96–189.38)	1	1999–2009	‒4.92 (−5.16 to −4.69)***	‒3.35 (−3.87 to −2.83)***
			2	2009–2018	‒2.78 (−3.16 to −2.39)***	
			3	2018–2021	1.41 (−2.26 to 5.22)	
			4	2021–2023	‒4.95 (−8.44 to −1.31)*	
South	3,736,179	187.21 (186.26–188.17)	1	1999–2002	‒3.30 (−4.81 to −1.76)***	‒3.40 (−3.93 to −2.87)***
			2	2002–2009	‒5.68 (−6.23 to −5.14)***	
			3	2009–2018	‒2.84 (−3.23 to −2.45)***	
			4	2018–2021	1.84 (−1.78 to 5.60)	
			5	2021–2023	‒5.62 (−8.99 to −2.13)**	
West	1,947,548	165.49 (164.33–166.66)	1	1999–2013	‒4.93 (−5.23 to −4.63)***	‒3.46 (−3.73 to −3.18)***
			2	2013–2023	‒1.35 (−1.9 to −0.8)***	

NH, not Hispanic; AAPC, average annual percentage change.

*p < 0.05; **p < 0.01; ***p < 0.001.

**Figure 2:**
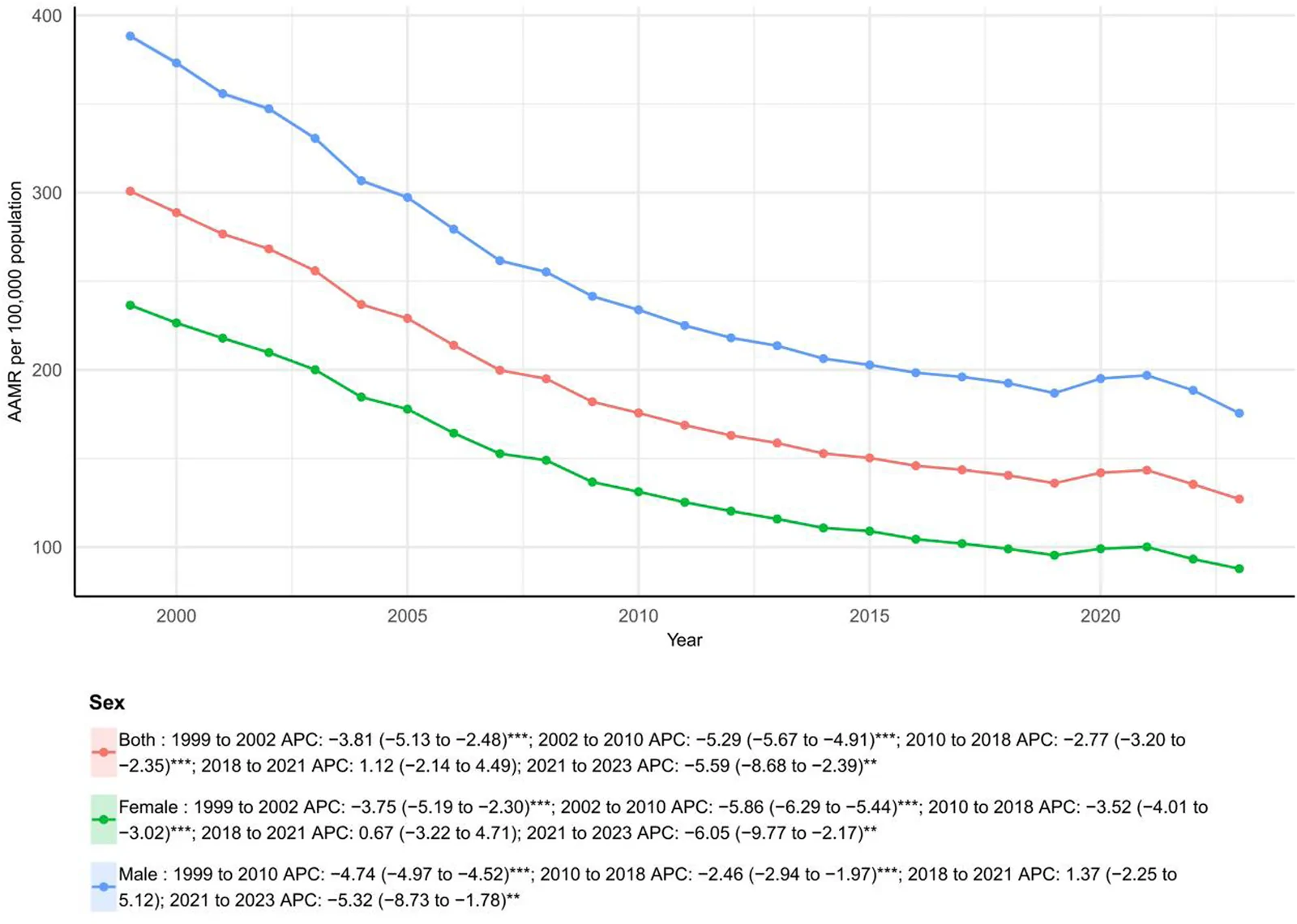
Overall and sex-stratified IHD-related AAMR per 100,000 population in the United States, 1999–2023.

### 3.2 Sex

Males accounted for 54.89% of total IHD deaths (*n* = 5,598,906), significantly exceeding females (45.11%, *n* = 4,601,456). Throughout the study period, male AAMR remained consistently higher than female AAMR: the overall average AAMR was 245.58 (95% CI: 244.55–246.62) for males and 138.69 (95% CI: 137.82–139.56) for females, with males having an approximately 1.8-fold higher AAMR. Both sexes exhibited phased, fluctuating downward trends in the AAMR (1999–2023), but declines were more pronounced among females (AAPC = −4.04, 95% CI: −4.61 to −3.47; *p* < 0.001) compared with males (AAPC = −3.29, 95% CI: −3.80 to −2.77; *p <* 0.001), with a 0.75 percentage point greater annual decline in females. A formal test of parallelism confirmed that the temporal trends differed significantly between the two sexes (*p* < 0.001; 4,500 permutations), indicating that the decline in IHD mortality was significantly steeper in females than in males (Table 2 and Supplementary Figure S1). A slight AAMR increase was observed for both sexes during 2018–2021, followed by the most substantial declines in the final phase (2021–2023): males (APC = −5.32, 95% CI: −8.73 to −1.78; *p <* 0.01) and females (APC = −6.05, 95% CI: −9.77 to −2.17; *p <* 0.01) (Figure 2 and Supplementary Tables S1, S3).

**Table 2:** Pairwise tests of parallelism for ischemic heart disease mortality trends by demographic and geographic subgroups.

Comparison	*K*_max_	Numerator df	Denominator df	Permutations	*p*-value	Result
Sex (1999–2023)						
Female vs. male	4	9	30	4,500	***	Rejected
Race/ethnicity (1999–2023)						
Hispanic vs. NH Black	4	9	30	4,500	**	Rejected
Hispanic vs. NH Other	2	5	38	4,500	**	Rejected
Hispanic vs. NH White	4	9	30	4,500	***	Rejected
NH Black vs. NH Other	4	9	30	4,500	**	Rejected
NH Black vs. NH White	4	9	30	4,500	*	Rejected
NH Other vs. NH White	4	9	30	4,500	0.3964	Failed to reject
Census region (1999–2023)						
Midwest vs. Northeast	4	9	30	4,500	***	Rejected
Midwest vs. South	4	9	30	4,500	***	Rejected
Midwest vs. West	4	9	30	4,500	***	Rejected
Northeast vs. South	4	9	30	4,500	**	Rejected
Northeast vs. West	4	9	30	4,500	*	Rejected
South vs. West	4	9	30	4,500	**	Rejected
Urbanization (1999–2020)						
Metropolitan vs. Non-metropolitan	3	7	28	4,500	***	Rejected

NH, not Hispanic; *K*_max_, assumed number of joinpoints for the comparison test; df, degrees of freedom. The maximum number of joinpoints (*K*_max_) for each pairwise test was determined based on the WBIC-optimal model of each subgroup, leading to varied *K*_max_ across comparisons. This software-driven setting should be considered when interpreting pairwise parallelism outputs due to differing joinpoint specifications between contrasts. Urbanization analysis limited to 1999–2020.

*p < 0.05; **p < 0.01; ***p < 0.001 by test of parallelism (4,500 permutations).

### 3.3 Racial/ethnic

Death counts varied by race/ethnicity: NH White individuals had the highest number (81.25%, *n* = 8,260,439), followed by NH Black (10.61%, *n* = 1,078,856), Hispanic (5.54%, *n* = 563,283), and NH Other (2.60%, *n* = 263,867). Significant differences in the AAMR were observed, with NH Black individuals having the highest AAMR [217.58 (95% CI: 215.49–219.68)], followed by NH White [190.10 (95% CI: 189.45–190.75)], Hispanic [141.90 (95% CI: 139.97–143.83)], and NH Other [109.60 (95% CI: 107.43–111.78)]. All racial/ethnic groups demonstrated significant long-term AAMR declines (all *p <* 0.001), with Hispanics showing the greatest reduction (AAPC = −4.13, 95% CI: −4.97 to −3.29), followed by NH Other (AAPC = −3.74, 95% CI: −4.30 to −3.19), NH Black (AAPC = −3.51, 95% CI: −3.91 to −3.10), and NH White (AAPC = −3.40, 95% CI: −4.02 to −2.77) (Figure 3 and Supplementary Tables S1, S4). Pairwise tests of parallelism revealed that all group comparisons reached statistical significance (all *p <* 0.05), with one exception: the difference in temporal trends between NH Other and NH White groups was not statistically significant (*p* = 0.3964) (Table 2 and Supplementary Figure S2).

**Figure 3:**
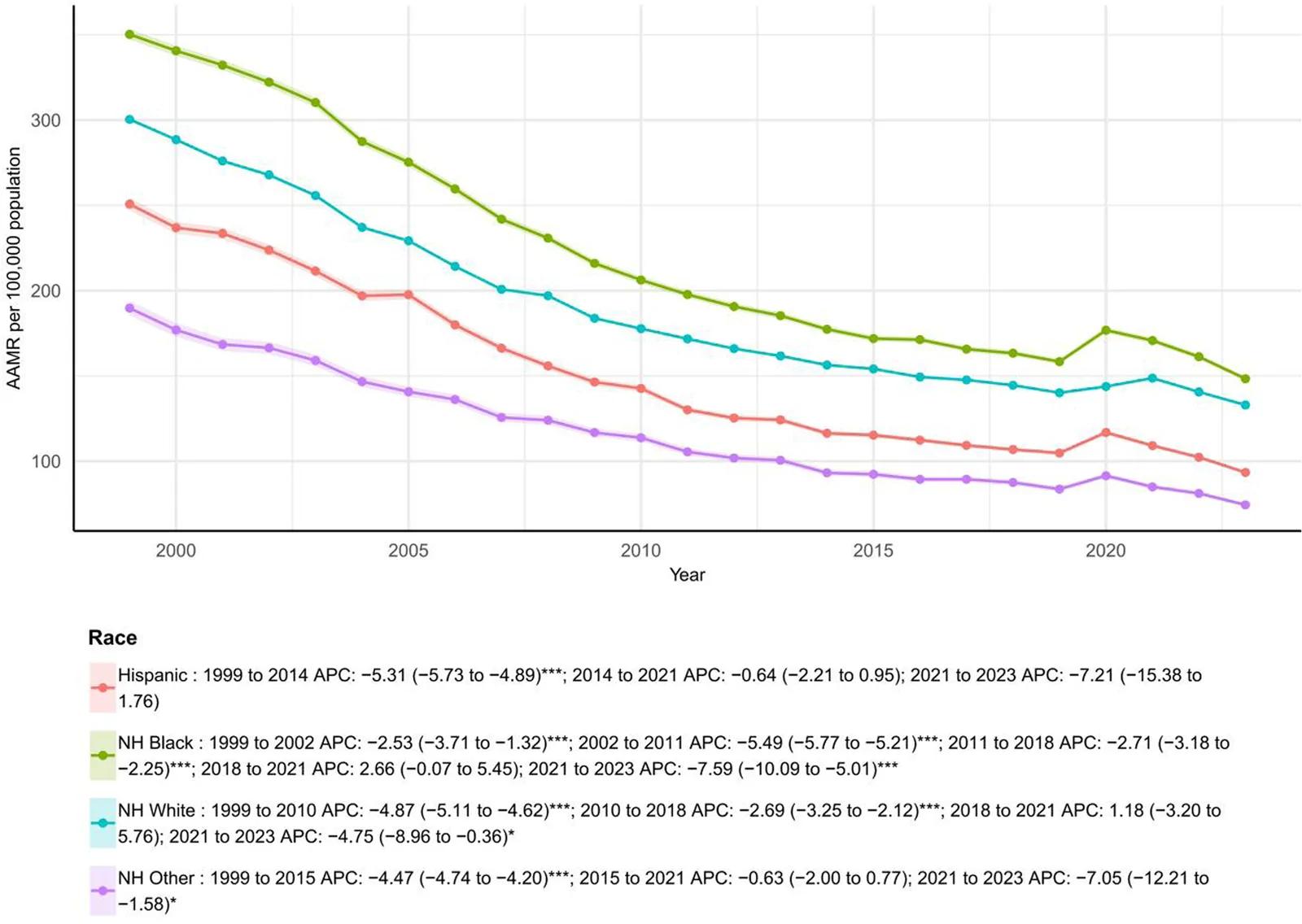
Race-stratified IHD-related AAMR per 100,000 population in the United States, 1999–2023.

The total number of deaths across the four mutually exclusive racial/ethnic subgroups was 10,166,445, approximately 33,917 fewer than the overall total of 10,200,362 IHD deaths included in this study. This discrepancy stems from records with unknown, unspecified, or missing racial/ethnic information extracted from CDC WONDER. Although these uncategorized deaths are incorporated into the overall aggregate mortality tally, they are excluded from stratified racial analyses in compliance with the cell suppression privacy protocols implemented within the CDC WONDER database.

### 3.4 Age

The CMR varied significantly across age groups, with a consistent “older age = higher CMR” pattern throughout the study period. In 1999, the highest CMR was observed in adults 85+ years [4,318.13 (95% CI: 4,298.15–4,338.12)] and the lowest in 25–34-year-olds [2.52 (95% CI: 2.37–2.68)]. By 2023, the CMR declined substantially across all age groups, but the age-related gradient persisted: 85+ years remained the highest [1,772.42 (95% CI: 1,761.94–1,782.90)] and 25–34 years the lowest [1.94 (95% CI: 1.81–2.06)]. Overall, the average CMR was 2,610.24 (95% CI: 2,596.80–2,623.68) for 85+ year and 2.37 (95% CI: 2.22–2.51) for 25–34-year-olds—a 1,101-fold difference. A joinpoint analysis revealed significant long-term declines in age-specific CMR across all age groups (all *p <* 0.05). The steepest decline was observed among adults aged 75–84 years (AAPC = −4.04; 95% CI: −4.16 to −3.94). In contrast, the youngest group (25–34 years) showed the smallest decline (AAPC = −0.89; 95% CI: −1.63 to −0.24) (Figure 4, Table 3, and Supplementary Table S5).

**Figure 4:**
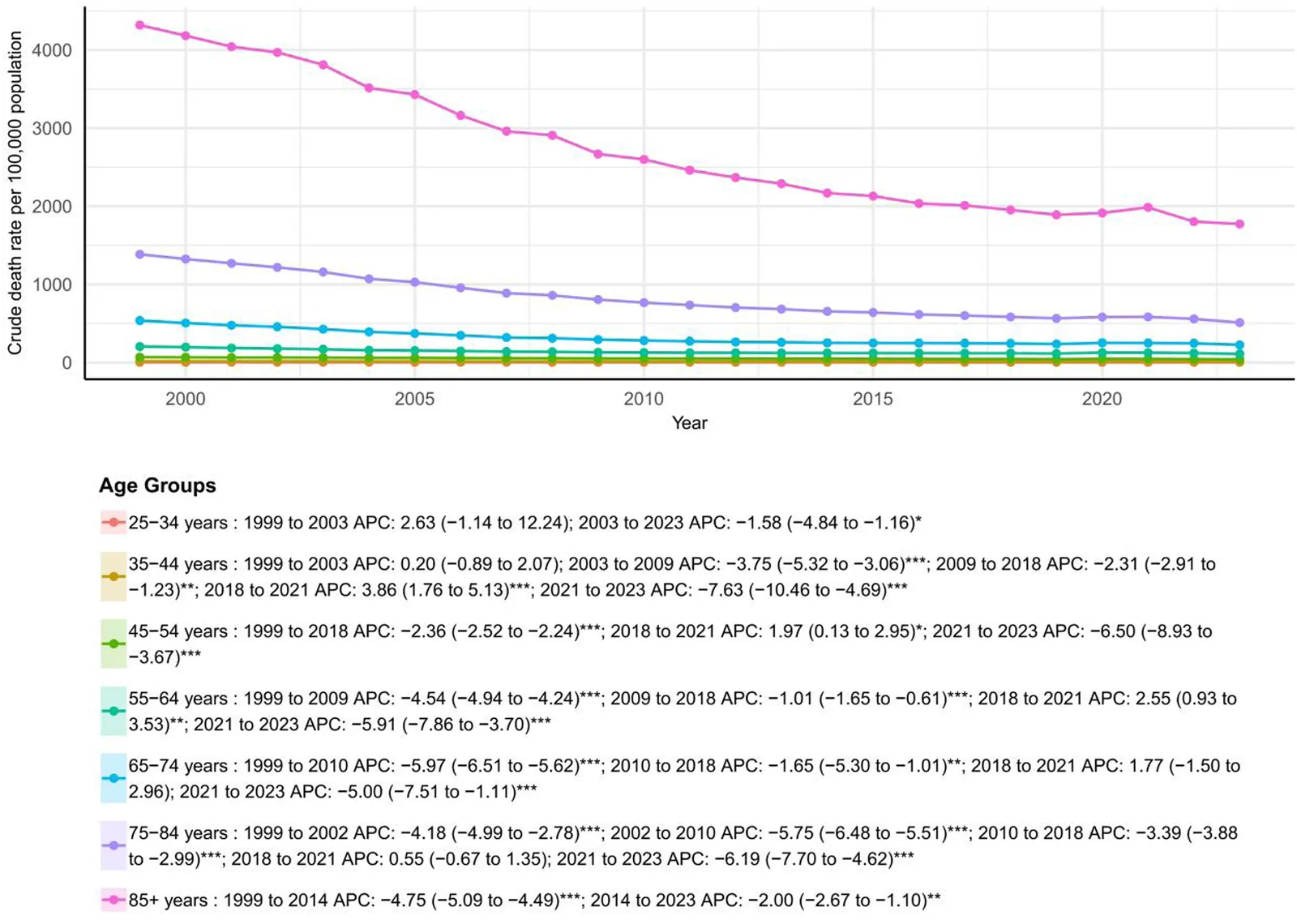
Age-stratified IHD-related CMR per 100,000 population in the United States, 1999–2023.

**Table 3:** IHD-related mortality among US adults (aged ≥25 years) (1999–2023): deaths, CMR, and APC.

Age	Deaths (*n*)	Overall CMR (95% CI)	Trend segment	Year interval	APC (95% CI)	AAPC (95% CI)
25–34 years	25,015	2.37 (2.22–2.51)	1	1999–2003	2.63 (−1.14 to 12.24)	‒0.89 (−1.63 to −0.24)*
			2	2003–2023	‒1.58 (−4.84 to −1.16)*	
35–44 years	145,451	13.69 (13.34–14.04)	1	1999–2003	0.20 (−0.89 to 2.07)	‒1.97 (−2.20 to −1.78)***
			2	2003–2009	‒3.75 (−5.32 to −3.06)***	
			3	2009–2018	‒2.31 (−2.91 to −1.23)**	
			4	2018–2021	3.86 (1.76 to 5.13)***	
			5	2021–2023	‒7.63 (−10.46 to −4.69)***	
45–54 years	546,327	52.07 (51.38–52.76)	1	1999–2018	‒2.36 (−2.52 to −2.24)***	‒2.19 (−2.38 to −2.06)***
			2	2018–2021	1.97 (0.13 to 2.95)*	
			3	2021–2023	‒6.50 (−8.93 to −3.67)***	
55–64 years	1,203,237	134.72 (133.51–135.92)	1	1999–2009	‒4.54 (−4.94 to −4.24)***	‒2.48 (−2.63 to −2.36)***
			2	2009–2018	‒1.01 (−1.65 to −0.61)***	
			3	2018–2021	2.55 (0.93 to 3.53)**	
			4	2021–2023	‒5.91 (−7.86 to −3.70)***	
65–74 years	1,845,522	301.26 (299.09–303.43)	1	1999–2010	‒5.97 (−6.51 to −5.62)***	‒3.52 (−3.72 to −3.37)***
			2	2010–2018	‒1.65 (−5.30 to −1.01)**	
			3	2018–2021	1.77 (−1.50 to 2.96)	
			4	2021–2023	‒5.00 (−7.51 to −1.11)***	
75–84 years	2,828,224	806.68 (802.01–811.35)	1	1999–2002	‒4.18 (−4.99 to −2.78)***	‒4.04 (−4.16 to −3.94)***
			2	2002–2010	‒5.75 (−6.48 to −5.51)***	
			3	2010–2018	‒3.39 (−3.88 to −2.99)***	
			4	2018–2021	0.55 (−0.67 to 1.35)	
			5	2021–2023	‒6.19 (−7.70 to −4.62)***	
85+ years	3,606,586	2,610.24 (2,596.80–2,623.68)	1	1999–2014	‒4.75 (−5.09 to −4.49)***	‒3.73 (−3.92 to −3.56)***
			2	2014–2023	‒2.00 (−2.67 to −1.10)**	

AAPC, average annual percentage change.

*p < 0.05; **p < 0.01; ***p < 0.001.

### 3.5 Urban–rural

Metropolitan areas had a significantly lower overall AAMR [189.07 (95% CI: 188.43–189.71)] compared with non-metropolitan areas [212.60 (95% CI: 211.12–214.08)]. Temporal trends differed by urbanization level: Non-metropolitan areas: rapid decline during 1999–2010 (APC = −4.26, 95% CI: −4.55 to −3.98; *p <* 0.01), followed by a slowed decline (2010–2020: APC = −1.72, 95% CI: −2.10 to −1.34; *p <* 0.01). Metropolitan areas: more granular decline phases with greater overall reduction: 1999–2002 (APC = −3.82, 95% CI: −5.11 to −2.52; *p <* 0.001), 2002–2010 (APC = −5.51, 95% CI: −5.89 to −5.14; *p <* 0.001), 2010–2018 (APC = −2.93, 95% CI: −3.34 to −2.51; *p <* 0.001), and 2018–2020 (stagnation, APC = 0.90, 95% CI: −2.29 to 4.20). Metropolitan areas had a higher long-term AAPC (−3.69, 95% CI: −4.06 to −3.33; *p <* 0.001) compared with non-metropolitan areas (−3.06, 95% CI: −3.28 to −2.84; *p <* 0.001) (Figure 5 and Supplementary Table S6). A formal test of parallelism confirmed that the temporal trends differed significantly between metropolitan and non-metropolitan areas (*p* < 0.001; 4,500 permutations) (Table 2 and Supplementary Figure S3). This analysis was confined to 1999–2020, as CDC WONDER data were available only for this time frame.

**Figure 5:**
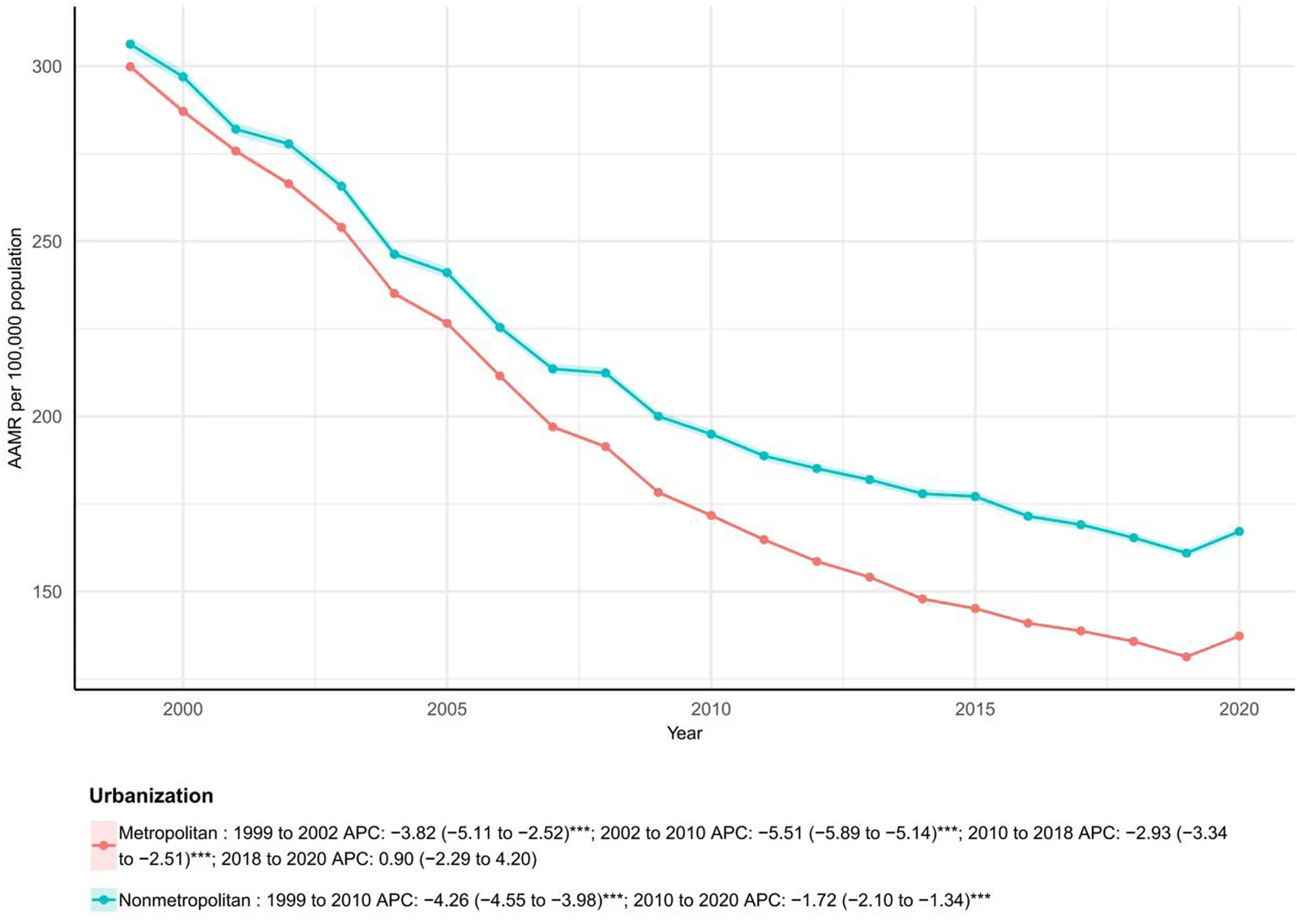
Urban–rural stratified IHD-related AAMR per 100,000 population in the United States, 1999–2020.

### 3.6 Geographic

#### 3.6.1 Region

Census regions exhibited distinct AAMR patterns and trends in IHD among US adults aged ≥25 years from 1999 to 2023. In terms of death counts, the South had the highest number of IHD deaths in 1999 (186,116), while the West had the lowest (95,464); by 2023, all regions recorded declines, with the Northeast experiencing the largest reduction (−47.18%, from 120,637 to 63,724) and the West the smallest (−24.83%, from 95,464 to 71,756), and the South remained the region with the highest death count (137,086).

With regard to AAMR levels, the Northeast had the highest overall AAMR [198.64 (95% CI: 197.32–199.96)], followed by the Midwest [188.17 (95% CI: 186.96–189.38)] and South [187.21 (95% CI: 186.26–188.17)], with the West the lowest [165.49 (95% CI: 164.33–166.66)]. In 1999, the Northeast maintained the highest AAMR (321.37, 95% CI: 319.56–323.19) and the West the lowest (276.05, 95% CI: 274.29–277.80); by 2023, the West remained the lowest (115.71, 95% CI: 114.86–116.57), while the Midwest became the region with the highest AAMR (132.81, 95% CI: 131.85–133.76). All regions showed significant long-term AAMR declines (all *p <* 0.001), with the Northeast having the largest AAPC (−3.86, 95% CI: −4.42 to −3.29), followed by the West (−3.50, 95% CI: −4.19 to −2.81), South (−3.40, 95% CI: −3.93 to −2.87), and Midwest (−3.35, 95% CI: −3.87 to −2.83). Pairwise tests of parallelism demonstrated that all six pairwise comparisons among the four regions were statistically significant (all *p <* 0.05), indicating heterogeneous temporal patterns in IHD mortality across US regions (Table 2 and Supplementary Figure S4).

Phase-specific APCs further revealed nuanced regional trends: the Northeast declined slowly from 1999 to 2002 (APC = −3.56, 95% CI: −5.06 to −2.03; *p <* 0.001), accelerated during 2002–2011 (APC = −5.19, 95% CI: −5.56 to −4.83; *p <* 0.001), slowed from 2011 to 2017 (APC = −2.98, 95% CI: −3.83 to −2.11; *p <* 0.001), plateaued in 2017–2020 (APC = −0.10, 95% CI: −4.06 to 4.03; *p <* 0.001), and reaccelerated in 2020–2023 (APC = −5.53, 95% CI: −7.50 to −3.51; *p <* 0.001); the Midwest declined rapidly from 1999 to 2009 (APC = −4.92, 95% CI: −5.16 to −4.69; *p <* 0.001), narrowed its decline from 2009 to 2018 (APC = −2.78, 95% CI: −3.16 to −2.39; *p <* 0.001), showed a slight upward trend in 2018–2021 (APC = 1.41, 95% CI: −2.26 to 5.22), and returned to a downward trajectory in 2021–2023 (APC = −4.95, 95% CI: −8.44 to −1.31; *p <* 0.05); the South declined slowly from 1999 to 2002 (APC = −3.30, 95% CI: −4.81 to −1.76; *p <* 0.001), entered a rapid decline phase from 2002 to 2009 (APC = −5.68, 95% CI: −6.23 to −5.14; *p <* 0.001), slowed from 2009 to 2018 (APC = −2.84, 95% CI: −3.23 to −2.45; *p <* 0.001), leveled off in 2018–2021 (APC = 1.84, 95% CI: −1.78 to 5.60), and accelerated again in 2021–2023 (APC = −5.62, 95% CI: −8.99 to −2.13; *p <* 0.01); the West declined rapidly from 1999 to 2013 (APC = −4.93, 95% CI: −5.23 to −4.63; *p <* 0.001) and declined again in 2013–2023 (APC = −1.34, 95% CI: −1.9 to −0.8; *p <* 0.001) (Figure 6 and Supplementary Table S7).

**Figure 6:**
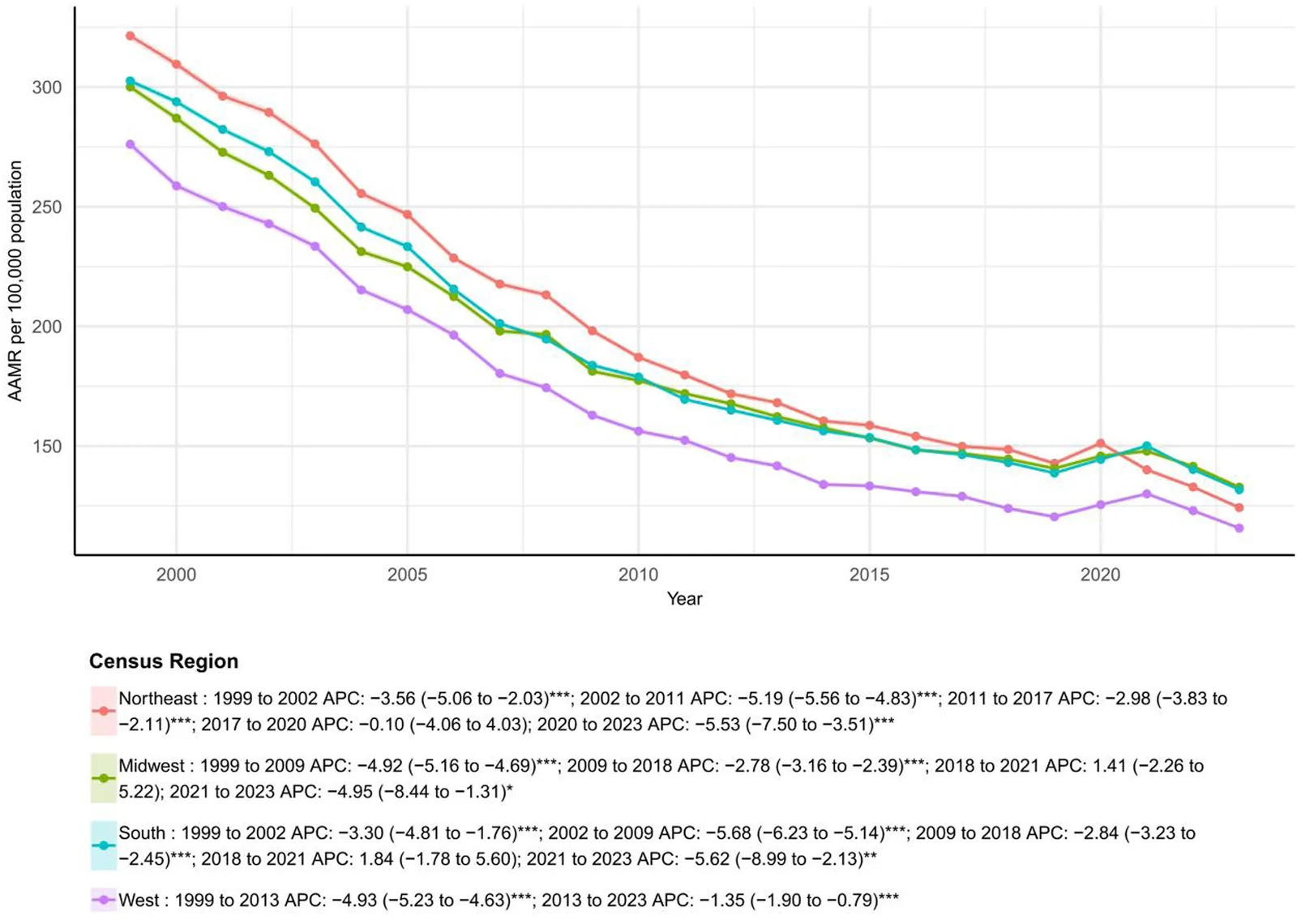
Region-stratified IHD-related AAMR per 100,000 population in the United States, 1999–2023.

#### 3.6.2 State

State-level AAMR distributions also shifted over the study period: overall, Oklahoma [260.99 (95% CI: 259.84–262.15)], New York [257.83 (95% CI: 257.35–258.31)], and Tennessee [246.35 (95% CI: 245.47–247.23)] had the highest AAMR, while Utah [121.69 (95% CI: 120.56–122.82)], Minnesota [122.59 (95% CI: 121.93–123.24)], and Hawaii [123.90 (95% CI: 122.61–125.18)] maintained the lowest, with the highest value approximately twice that of the lowest. Notably, 2023 AAMR data revealed a hierarchical pattern: states in the 90th percentile—including Arkansas [182.96 (95% CI: 177.59–188.33)], Tennessee [178.11 (95% CI: 174.56–181.66)], West Virginia [177.08 (95% CI: 170.59–183.56)], Michigan [167.61 (95% CI: 164.83–170.39)], and Mississippi [167.08 (95% CI: 161.75–172.41)]—had AAMR approximately 1.6 times higher than those in the 10th percentile, which included Colorado [89.41 (95% CI: 86.52–92.30)], Minnesota [90.20 (95% CI: 87.47–92.93)], Connecticut [92.10 (95% CI: 88.78–95.43)], Massachusetts [92.79 (95% CI: 90.33–95.25)], and Alaska [93.97 (95% CI: 84.84–103.10)] (Figure 7 and Supplementary Tables S8, S9).

**Figure 7:**
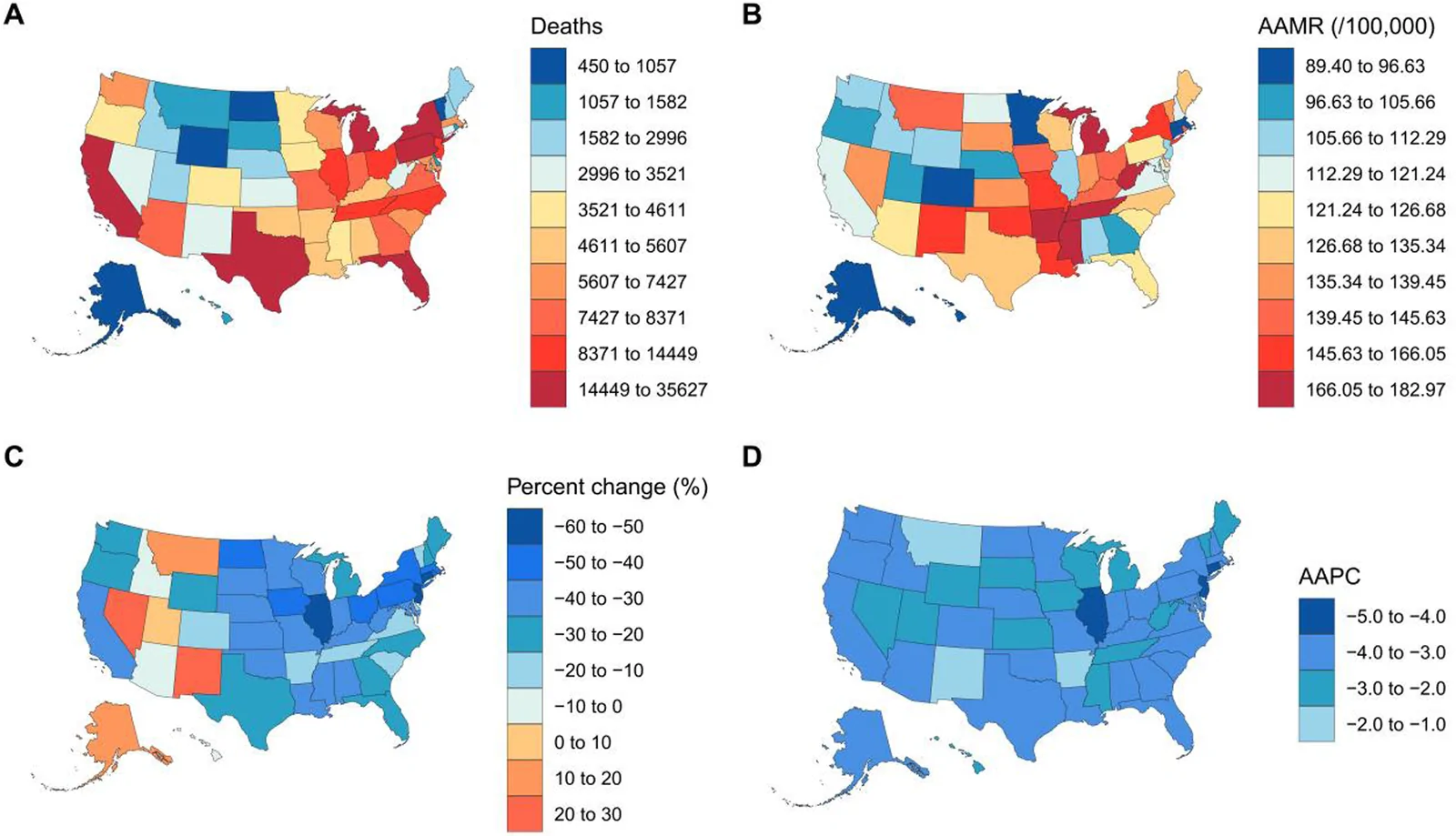
State-stratified change related to IHD in the United States, 1999–2023 in total deaths **(A)**, AAMR per 100,000 population **(B)**, percent changes **(C)**, and AAPC **(D)**.

## 4 Discussion

Using mortality data from the CDC WONDER database, this study investigated IHD mortality trends and disparities among US adults aged ≥25 years between 1999 and 2023. Key findings revealed an overall downward trend in IHD mortality—with the AAMR declining from 300.8 to 127.07 per 100,000 population (AAPC = −3.51; *p <* 0.001)—yet, there were persistent disparities across age, race/ethnicity, sex, and geographic regions. The temporal trend exhibited a “fast-slow-fast” fluctuating pattern, with the most rapid decline observed during 2021–2023 (APC = −5.59; *p <* 0.01). Sex-specific analyses showed that male AAMR (overall average: 245.58) was approximately 1.8-fold higher than female AAMR (138.69), but females experienced a greater long-term annual decline (AAPC = −4.04 vs. −3.29 for males), with more pronounced reductions across all declining phases. Racial/ethnic disparities persisted: NH Black individuals had the highest AAMR (217.58), while NH Other groups had the lowest (109.60), and all racial/ethnic groups demonstrated significant declines, with Hispanics showing the largest reduction (AAPC = −4.13). Age was strongly associated with CMR, with rates increasing exponentially with age—peaking among adults 85+ years (2,610.24) and lowest among 25–34-year-olds (2.37)—and all age groups except 25–34-year-olds recording significant declines, with the steepest reduction in 75–84-year-olds (AAPC = −4.04). Geographically, the Northeast had the highest overall AAMR (198.64) and the largest long-term decline (AAPC = −3.86), while the West maintained the lowest AAMR (165.49). Metropolitan areas exhibited a greater long-term decline (AAPC = −3.69) than non-metropolitan areas (AAPC = −3.06). At the state level, Oklahoma had the highest AAMR (260.99) and Utah the lowest (121.69) in 2023.

As a descriptive population surveillance analysis, our study delivers three key novel insights compared with prior relevant work (9). First, we adopt a broader adult population cutoff of ≥25 years rather than restricting analyses to older adults aged ≥75 years, enabling a comprehensive assessment of full age-based IHD mortality disparities. Second, we extended surveillance data to 2023 to characterize the postpandemic mortality recovery trend unavailable in studies limited to 1999–2020. Third, we implemented formal pairwise parallelism tests across all demographic and geographic subgroups to quantify slope heterogeneity, a quantitative analytical strategy absent from previous related analyses.

Unlike previous analyses limited to the prepandemic period (e.g., 1999–2020), our study extends through 2023 and reveals a distinct postpandemic recovery phase (2021–2023) characterized by the steepest declines observed across the 25-year study period (overall APC = −5.59). This acceleration was not uniform across subgroups: recovery was significantly slower among males, NH Black individuals, suggesting that the pandemic may have widened pre-existing disparities.

Formal pairwise comparisons suggest significant heterogeneity in IHD mortality trends across all demographic and geographic subgroups. Trend parallelism was rejected between males and females (*p <* 0.01), consistent with known sex disparities in cardiovascular mortality decline. Similar significant differences were observed among the four census regions and between urban and rural areas, underscoring the sustained impact of geographic factors on IHD mortality. Pairwise tests further revealed divergent temporal patterns across most racial/ethnic groups (*p <* 0.05). The only non-significant comparison was between NH Other and NH White individuals (*p* = 0.3964), likely due to high heterogeneity within the NH Other group, which includes Asians, American Indians/Alaska Natives, and multiracial populations with distinct cardiovascular risk profiles. Collectively, these results confirm that IHD mortality trends are not uniform across US subgroups, supporting the need for targeted, subgroup-specific prevention strategies.

The overall downward trend in IHD mortality aligns with improvements in economic development and medical care in the United States, as well as enhanced public health awareness of modifiable risk factors (e.g., dietary quality and physical activity) (13, 14). This finding is consistent with that of prior research demonstrating an inverse association between national development level and IHD burden (15). Notably, a transient increase in mortality during 2018–2021 coincided with the COVID-19 pandemic, which disproportionately affected individuals with pre-existing cardiovascular conditions—20%–35% of COVID-19-related deaths occurred in this population (16), with IHD being the most common underlying cardiovascular disease (17). COVID-19 exacerbated IHD mortality by increasing complications such as myocardial injury (27.8%) and heart failure (23%) (18, 19) and straining healthcare resources (20). This may be directly related to cardiovascular complications induced by SARS-CoV-2 infection, including myocardial injury and thrombotic events (21). During the pandemic period in the United States, indirect effects arose from disrupted medical services, such as delayed routine care and delayed emergency treatment for patients with acute coronary syndrome, raising public concerns. Notably, the sharp mortality decline observed from 2021 to 2023 may partly reflect a postpandemic restoration of cardiovascular healthcare services. Nevertheless, this trend could also be driven in part by mortality displacement (the “harvesting effect”): many high-risk individuals with pre-existing IHD died prematurely during the 2020–2021 COVID-19 surge, leaving a smaller pool of susceptible individuals in subsequent years. Formal counterfactual modeling would be needed to separate these two effects.

Sex disparities in IHD mortality—with females showing greater declines despite historically lower baseline rates—are consistent with the established literature on cardiovascular disease sex differences (19). Estrogen's cardioprotective effects, including endothelium-dependent vasodilation, favorable lipoprotein metabolism, and inhibition of vascular smooth muscle proliferation (22–25), may contribute to lower female mortality. In addition, improved diagnostic rates and treatment access for women in recent years—addressing the issue of historical underdiagnosis and prehospital delay (26–28)—likely explain the more pronounced mortality reductions observed.

Racial/ethnic disparities, particularly the persistently highest AAMR among NH Black individuals, are rooted in systemic inequities. Barriers to advanced treatments (e.g., microaxial left ventricular assist devices and extracorporeal membranous oxygenation) (29), lower insurance coverage rates (30), and reduced acceptance of aggressive interventions contribute to these disparities, highlighting the need to address the structural determinants of health (31).

Age-related increases in CMR reflect the cumulative impact of aging on cardiovascular health. Mitochondrial dysfunction, autophagic impairment, and vascular aging (e.g., arterial stiffening and endothelial dysfunction) increase IHD risk in older adults (32). Comorbidities such as dyslipidemia, hypertension, and type 2 diabetes—prevalent in older populations—further worsen prognosis (33, 34), while polypharmacy and atypical symptom presentation may delay diagnosis and treatment (9). Limited treatment options for elderly individuals (e.g., high surgical risk for coronary artery bypass grafting (CABG) and age-related graft vessel dysfunction) also contribute to higher mortality (35). However, this study also demonstrated that the group with the largest reduction in mortality was adults aged 75–84 years, rather than adults aged 85 years and above. The rapid mortality decline observed in the younger elderly population is closely associated with advances in contemporary medical care. Invasive interventions, including percutaneous coronary intervention (PCI) and CABG, have been more widely adopted in this younger elderly subgroup (36), whereas patients aged 85 years and above are more likely to receive conservative management because of elevated perioperative and procedural risks. In addition, the magnitude of mortality reduction was markedly smaller among individuals aged 25–34 years. Although the relatively small at-risk population in this age group is one contributing factor, a concerning trend warrants attention: the rising prevalence of obesity, diabetes, and physical inactivity among younger birth cohorts may be attenuating the population-level downward trend in IHD mortality (37, 38).

Geographic disparities persist across urban–rural and regional contexts. Metropolitan areas had lower AAMRs and faster declines than non-metropolitan areas, which may be partly attributed to better healthcare accessibility, availability of specialized facilities, and higher socioeconomic status (e.g., income and education) (39). The transient 2019 mortality increase in both settings was linked to COVID-19-related cardiovascular disease exacerbations. Regionally, the West maintained the lowest AAMR potentially related to superior healthcare infrastructure and favorable socioeconomic factors (39), while the Northeast—historically the highest—experienced a significant post-2021 decline, shifting the highest regional AAMR to the Midwest by 2023. This trend may reflect substantial investments in medical resources within the Northeast region, including the enhancement of healthcare infrastructure and the implementation of targeted prevention and treatment initiatives for cardiovascular diseases. State-level differences were striking: Arkansas, Mississippi, and Tennessee had high AAMs associated with unfavorable risk profiles [e.g., elevated systolic blood pressure, fasting glucose, low-density lipoprotein cholesterol (LDL-C), smoking prevalence, and low cereal intake] (40), while Minnesota exhibited lower cardiovascular mortality and healthier metabolic markers (41, 42).

In conclusion, while in the United States, IHD mortality has declined overall, persistent disparities across demographic and geographic subgroups underscore the influence of socioeconomic factors, healthcare accessibility, and lifestyle patterns. Future research should prioritize identifying the underlying determinants of these disparities to inform targeted interventions, reduce health inequities, and improve population-level IHD outcomes.

## 5 Limitations

This study has several limitations. First, the CDC WONDER database did not include 2024–2025 data at the time of analysis, precluding the examination of the most recent IHD mortality trends; future investigations should incorporate these data once available.

Second, the study relied exclusively on CDC WONDER data derived from death certificates, which are subject to inherent biases. Potential sources of error include ambiguous classification of patient subgroups, reliance on death certificate diagnoses without definitive antemortem confirmation, misdiagnosis, and inaccurate coding of ICD codes by data entry personnel. These factors may lead to an underestimation or misclassification of IHD-related deaths, thereby affecting the accuracy and reliability of the findings. Future research should validate results using multiple complementary data sources.

Third, CDC WONDER lacks detailed clinical information, including patient comorbidities, diagnostic and therapeutic strategies, clinical examination results, and laboratory indicators. This precluded a more comprehensive analysis of IHD mortality determinants and limited the depth of insight into modifiable and non-modifiable risk factors in this study. Furthermore, CDC WONDER lacks county-level socioeconomic and healthcare-access metrics, precluding age-period cohort modeling and formal mechanistic testing. The aggregated “NH Other” category includes American Indian/Alaska Native, Asian American, Native Hawaiian/Pacific Islander, and multiracial subgroups. CDC WONDER updated its race coding after 2020; to maintain consistent grouping across 1999–2023, we kept this pooled category. Disaggregated subgroup data are often suppressed by cell-suppression rules because of small death counts, and we used the four-group race ethnicity classification common in CDC-WONDER-based studies (43). More multisource linked datasets are needed in the future to explore the causal drivers of mortality disparities.

Fourth, we conducted only one-dimensional stratified analyses but did not assess potential interactions among these factors. For instance, NH Black men living in non-metropolitan areas may bear compound mortality risks that single-axis stratification cannot detect. Future work could construct multiway interaction Poisson regression models to quantify joint subgroup risks, although detailed intersectional breakdowns are constrained by the small-cell suppression policy of the CDC WONDER database. Intersectional multilevel regression models are therefore recommended for follow-up research to disentangle joint subgroup risks.

Fifth, death certificate coding bias existed throughout the COVID-19 period (2020–2021). Many patients with pre-existing IHD were recorded with COVID-19 as the primary underlying cause of death, which may slightly underestimate real IHD mortality and partly explain the mild mortality uptick from 2018 to 2021. Furthermore, the rapid 2021–2023 IHD mortality decline may be partly influenced by COVID-19-related mortality-displacement (harvesting) effects, which cannot be quantified in the present analysis.

Sixth, there is a constraint that is exclusive to urban–rural subgroup analyses: urbanicity classification data in CDC WONDER are available only up to 2020. Therefore, we cannot stratify our core novel finding—the sharp postpandemic IHD mortality decline during 2021–2023–by metropolitan or non-metropolitan residence. We are unable to compare disparities in the pace and magnitude of postpandemic mortality recovery between urban and rural populations; however, this data gap does not apply to other stratified dimensions of our study.

## 6 Conclusion

This study investigated IHD mortality trends and disparities in US adults aged ≥25 years using CDC WONDER data (1999–2023). Despite an overall downward trend in IHD mortality (AAMR decline: 300.8–127.07 per 100,000; AAPC = −3.51; *p <* 0.05), significant disparities persist across sex, race/ethnicity, age, and geographic regions. These inequities are particularly pronounced among males, NH Black individuals, and older adults in the Midwest. In summary, the findings underscore the complex interplay of socioeconomic, clinical, and structural factors contributing to IHD mortality disparities, highlighting the urgent need for targeted interventions by governmental and public health authorities. This study provides actionable guidance for policymakers to develop evidence-based programs aimed at addressing healthcare inequities, enhancing disease surveillance systems, and optimizing integrated care strategies for high-risk populations. Such measures are critical to reducing persistent disparities and improving population-level IHD outcomes.

## Data Availability

The original contributions presented in the study are included in the article/Supplementary Material, and further inquiries can be directed to the corresponding authors.
