# Supplementary material for: Ischemic heart disease mortality trends and disparities in US adults from 1999 to 2023: a CDC WONDER analysis
Source: Front Cardiovasc Med. 2026 Sep 18;13:1883973. doi: 10.3389/fcvm.2026.1883973 (PMC13630521; doi:10.3389/fcvm.2026.1883973)
Supplement: Supplementary file 1 [file Datasheet1.docx]

Supplementary Figure S1. Pairwise comparison of sex-specific ischemic heart disease (IHD)-related age-adjusted mortality rate (AAMR) trends, 1999-2023.

Final selected models: female, 4 joinpoints; male, 3 joinpoints. Test of parallelism rejected the null hypothesis of equal trends (P = 0.0002; 4,500 permutations).

### Supplementary Figure S2. Pairwise comparisons of ischemic heart disease(IHD)-related age-adjusted mortality rate (AAMR) trends by race/ethnicity, 1999–2023.

(a) Hispanic individuals (2 joinpoints) vs. Non-Hispanic Black individuals (4 joinpoints).(b) Hispanic individuals (2 joinpoints) vs. Non-Hispanic Other individuals (2 joinpoints).(c) Hispanic individuals (2 joinpoints) vs. Non-Hispanic White individuals (3 joinpoints).(d) Non-Hispanic Black individuals (4 joinpoints) vs. Non-Hispanic Other individuals (2 joinpoints).(e) Non-Hispanic Black individuals (4 joinpoints) vs. Non-Hispanic White individuals (3 joinpoints).(f) Non-Hispanic Other individuals (4 joinpoints) vs. Non-Hispanic White individuals (4 joinpoints).

Tests of parallelism rejected equal trends for all pairwise comparisons except for the comparison in panel (f) (P>0.05; see Table 3 for full statistical details).*APC significantly different from zero (p < 0.05).

### Supplementary Figure S3. Pairwise comparison of ischemic heart disease (IHD)-related age-adjusted mortality rate (AAMR) trends by urban-rural status, 1999–2020.

(a) Metropolitan areas (3 joinpoints) versus Nonmetropolitan areas (1 joinpoint).

The test of parallelism rejected equal trends between the two groups (P < 0.05; see Table 3 for full statistical details).*APC significantly different from zero (p < 0.05).

Supplementary Figure S4. Pairwise comparisons of ischemic heart disease(IHD)-related age-adjusted mortality rate (AAMR) trends by census region,1999-2023.

(a) Midwest (3 joinpoints) vs. Northeast (4 joinpoints). (b) Midwest (3 joinpoints)vs. South (4 joinpoints). (c) Midwest (3 joinpoints) vs. West (4 joinpoints). (d)Northeast (4 joinpoints) vs. South (4 joinpoints). Tests of parallelism rejected equal trends for all pairwise comparisons (all P < 0.05; see Table 3 for full statistical details). \*APC significantly different from zero (p < 0.05).
